# Development of high-titer polyclonal antisera targeting a local bovine viral diarrhea virus-1a strain: A preliminary study from Indonesia

**DOI:** 10.14202/vetworld.2025.3208-3217

**Published:** 2025-10-31

**Authors:** S. U. Khan, H. Wuryastuty, M. H. Wibowo, R. Wasito, D. Aji, Sarmin Sarmin

**Affiliations:** 1 Department of Microbiology, University of Rasul, Mandi Bahaud Din, Punjab, Pakistan; 2 Department of Veterinary Internal Medicine, Faculty of Veterinary Medicine, Gadjah Mada University, Yogyakarta, Indonesia; 3 Department of Veterinary Microbiology, Faculty of Veterinary Medicine, Gadjah Mada University, Yogyakarta, Indonesia; 4 Department of Veterinary Pathology, Faculty of Veterinary Medicine, Gadjah Mada University, Yogyakarta, Indonesia; 5 Department of Surgery and Radiology, Faculty of Veterinary Medicine, Gadjah Mada University, Yogyakarta, Indonesia; 6 Department of Veterinary Physiology, Faculty of Veterinary Medicine, Gadjah Mada University, Yogyakarta, Indonesia

**Keywords:** bovine viral diarrhea virus-1a, diagnostic tool, enzyme-linked immunosorbent assay, immunoperoxidase assay, Indonesia, polyclonal antisera

## Abstract

**Background and Aim:**

Bovine viral diarrhea virus (BVDV) is an economically significant pathogen of cattle, causing reproductive disorders, immunosuppression, and production losses worldwide. In Indonesia, BVDV-1a is among the most prevalent subgenotypes; however, field diagnosis still relies heavily on imported kits developed using non-local strains, which can lead to potential gaps in sensitivity and specificity. Locally tailored immunological reagents could enhance diagnostic accuracy and support future control strategies. This study aimed to produce and characterize polyclonal antisera against a local BVDV-1a isolate from Indonesia.

**Materials and Methods:**

Four New Zealand white rabbits were immunized with inactivated BVDV-1a antigen propagated in Madin–Darby bovine kidney (MDBK) cells. Booster immunizations were administered on days 14 and 28. Ten days after the final booster, sera were collected, pooled, and purified using ammonium sulfate precipitation and dialysis. Purified antisera were characterized by sodium dodecyl sulfate–polyacrylamide gel electrophoresis (SDS-PAGE). Antibody titers were assessed using indirect enzyme-linked immunosorbent assay (ELISA), and specificity was validated by immunoperoxidase monolayer assay (IPMA) in infected MDBK cells.

**Results:**

The purification process yielded polyclonal antisera with a protein concentration of 40.33 mg/mL. SDS-PAGE revealed characteristic bands at approximately 53, 75, and 100 kDa, consistent with immunoglobulin components. Indirect ELISA showed strong antibody titers, with positive reactivity sustained up to 1:100. IPMA confirmed specific recognition of BVDV antigens, as infected MDBK cells exhibited distinct cytoplasmic staining, whereas uninfected controls remained negative.

**Conclusion:**

This preliminary study successfully generated high-titer polyclonal antisera against a local Indonesian BVDV-1a strain. The antibodies demonstrated robust reactivity and specificity, highlighting their potential utility as foundational reagents for developing regionally relevant diagnostic assays. While limited by a small sample size and pooled sera, these findings represent an important first step toward establishing locally adapted immunodiagnostic resources for BVDV. The development of local diagnostic tools not only strengthens veterinary disease surveillance but also safeguards livestock-dependent livelihoods, enhances food security, and reduces reliance on imported kits. Improved BVDV control in cattle contributes to One Health by minimizing economic losses, ensuring the safety of animal-derived food products, and reducing the risk of viral persistence in mixed livestock populations.

## INTRODUCTION

Bovine viral diarrhea virus (BVDV), a member of the genus *Pestivirus* within the family *Flaviviridae*, is a highly contagious pathogen of cattle with major implications for animal health and productivity. Infection can present with a wide clinical spectrum, ranging from subclinical to severe disease, and impacts multiple physiological systems, including reproductive, digestive, and immune functions. Consequently, BVDV causes significant economic losses in the cattle industry worldwide through reproductive disorders, reduced herd performance, and elevated veterinary costs [1, 2].

The *Pestivirus* genus comprises four recognized species: BVDV-1 *(Pestivirus A)*, BVDV-2 *(Pestivirus B)*, classical swine fever virus *(Pestivirus C)*, and border disease virus *(Pestivirus D)*. Sequence-based analyses further subdivide BVDV-1 into 21 subgenotypes (BVDV-1a to BVDV-1u) and BVDV-2 into 4 (BVDV-2a to BVDV-2d). Among these, BVDV-1a is one of the most widespread subgenotypes and is particularly prevalent in Indonesia [3–5]. This high genetic variability complicates both vaccine development and diagnostic accuracy, as antigenic differences among strains influence immune responses and test performance [6].

Global strategies for BVDV control and eradication emphasize three pillars: (1) Reliable, rapid, and cost-effective diagnostic methods for identifying acute and persistent infections; (2) efficacious vaccines tailored to circulating strains; and (3) strict biosecurity measures [7, 8]. These measures are essential because BVDV demonstrates notable environmental resilience, making eradication from infected herds especially challenging [9]. The effectiveness of control programs, therefore, hinges on precise and context-specific diagnostic capacity.

Diagnostic approaches for BVDV typically rely on antibody-based assays such as enzyme-linked immunosorbent assay (ELISA), immunohistochemistry (IHC), and virus neutralization tests, all of which require high-quality antisera. ELISA remains the most widely used tool for surveillance [10], especially in unvaccinated herds, where antibodies signal natural exposure. However, commercial ELISA kits often face limitations, including false positives and negatives due to antigenic cross-reactivity or non-specific binding [11, 12]. Diagnostic accuracy is further reduced when kits are based on non-local viral strains, a significant issue in Indonesia, where BVDV-1a predominates but field diagnosis relies heavily on imported ELISA kits [3, 4].

At present, Indonesia lacks a comprehensive national BVDV control program [13]. The absence of standardized diagnostic protocols and locally produced immunological reagents poses a major obstacle to surveillance and disease management. A recent study by Nugroho *et al*. [14] confirms BVDV endemicity in at least 10 of 34 provinces, yet the virus remains absent from Indonesia’s official priority disease list. Field surveys in imported cattle using commercial kits have further highlighted the virus’s distribution, underscoring the urgent need for locally adapted diagnostic tools [15]. Without region-specific antisera, diagnostic precision is compromised, hindering both surveillance and the development of effective, evidence-based control measures.

Despite the recognized economic and health impacts of BVDV, particularly the BVDV-1a subgenotype that is prevalent in Indonesia, research and control programs in the country remain limited. The current diagnostic efforts heavily rely on commercial ELISA kits developed using viral strains from outside Indonesia, which may not accurately detect local variants due to antigenic differences. This reliance increases the risk of false-positive and false-negative results, ultimately undermining surveillance accuracy and disease management strategies. Furthermore, there is a lack of standardized diagnostic protocols and region-specific immunological reagents, leaving veterinarians and policymakers with inadequate tools for timely detection and control. Although global efforts emphasize the importance of tailored diagnostic resources, few studies in Indonesia have focused on generating polyclonal antisera or other reagents that target locally circulating BVDV strains. Consequently, the gap between the availability of commercial diagnostic kits and the need for regionally relevant tools persists, restricting the capacity to establish effective national control or eradication programs.

This preliminary study was designed to address a critical gap by producing and characterizing polyclonal antisera against a locally isolated BVDV-1a strain from Indonesia. By immunizing rabbits with the inactivated viral antigen and purifying the resulting sera, the study aimed to evaluate the antibody yield, purity, and specificity using standard assays, including ELISA, sodium dodecyl sulfate–polyacrylamide gel electrophoresis (SDS-PAGE), and the immunoperoxidase monolayer assay (IPMA). The overarching aim was to provide a foundational resource for the development of region-specific diagnostic reagents that could improve the accuracy of BVDV detection in Indonesia. In the longer term, these reagents may serve as essential tools to strengthen disease surveillance, guide epidemiological studies, and support the formulation of future government-led BVDV control and eradication programs in line with international One Health strategies.

## MATERIALS AND METHODS

### Ethical approval

The study was conducted between March and December 2023 at the Laboratory of Veterinary Internal Medicine, Faculty of Veterinary Medicine, Gadjah Mada University, Yogyakarta, Indonesia. Four male New Zealand white rabbits (5 months old; ~2.5 kg) were used for polyclonal antisera production. Ethical approval was obtained from the Ethical Clearance Commission of Gadjah Mada University (Approval No. 045/EC-FKH/Int/2023).

### Study period and location

The experiment was conducted from March to December 2023 at the Laboratory of Veterinary Internal Medicine, Faculty of Veterinary Medicine, Gadjah Mada University, Yogyakarta, Indonesia.

### Animal husbandry

Rabbits were individually housed in stainless steel cages equipped with automatic water bottles and pelleted diet feeding boxes. The animal facility was maintained at 24°C under a 12:12 h light–dark cycle. Animals were provided with a commercial pelleted diet and clean *ad libitum* drinking water.

### Virus source and propagation

A local field isolate of BVDV-1a was obtained from previously confirmed positive cattle samples in Yogyakarta, Indonesia, and identified by a reverse transcription-polymerase chain reaction (RT-PCR), E2 region sequencing, and genetic analysis [16]. The virus was propagated in Madin–Darby bovine kidney (MDBK) cells cultured in Eagle’s minimum essential medium (EMEM) supplemented with 10% fetal bovine serum (FBS), 100 U/mL penicillin, and 100 μg/mL streptomycin. Successful propagation was confirmed by observation of cytopathic effect (CPE) and RT-PCR.

### Antigen preparation

The viral concentration in the culture supernatant was determined using a modified tissue culture infectious dose 50% and plaque assay [17], as the isolate did not induce visible plaques or CPE in MDBK cells. MDBK cells (3 × 10^5^ cells/mL) were seeded into 96-well plates (50 μL/well plus 100 μL growth medium) and incubated at 37°C with 5% CO_2_ until ~80% confluence. Tenfold serial dilutions (10^−2^–10^−7^) of the virus stock were inoculated (100 μL/well, four replicates per dilution). After adsorption for 2 h, 150 μL infection medium was added, and the plates were incubated for 4 days. The viral endpoint was defined by consistent IHC results across three evaluations. Ultraviolet (UV) spectrophotometry (Shimadzu, Tokyo, Japan) quantified the harvested supernatant at 0.513 mg/mL.

Supernatants were collected 3–5-days post-infection and clarified by centrifugation (3,000 × *g*, 10 min). Viral inactivation was achieved with 0.1% (v/v) β-propiolactone (BPL) at 4°C for 24 h, followed by incubation at 37°C for 2 h to hydrolyze residual BPL. Inactivation was confirmed by three blind passages in MDBK cells, showing no CPE or positive PCR results [18,19].

### Rabbit immunization and serum collection

Each rabbit received 500 μL of antigen solution (500 μg/mL in PBS [pH 7.2] emulsified 1:1 with complete Freund’s adjuvant) administered subcutaneously at four sites. Clinical signs and body temperature were monitored daily post-immunization. Booster injections were given on days 14 and 28 using antigen diluted to 200 μg/mL in PBS and emulsified with incomplete Freund’s adjuvant [20–23].

Ten days after the final booster, 5 mL of blood was collected from the femoral vein. Samples were clotted at room temperature (3 h) and centrifuged at 1,000 × *g* for 20 min. Serum was transferred to sterile microtubes (Thermo Fisher Scientific, Waltham, MA, USA), heat-inactivated at 56°C for 30 min [24], and stored at −20°C. To minimize inter-animal variability, sera from all rabbits were pooled for the preparation of polyclonal antisera [25].

### Purification of polyclonal antisera

Rabbit sera were purified using ammonium sulfate (AMS) precipitation [26]. Serum was brought to 40% saturation with solid AMS under constant stirring at 4°C, incubated overnight, and centrifuged (1,000 × *g*, 20 min). The pellets were resuspended in PBS (pH 7.2), and the precipitation process was repeated to increase purity. The antibody fraction was dialyzed against PBS (pH 7.2) using a Mega D-Tube dialyzer (Merck KGaA, Darmstadt, Germany) membrane (6–8 kDa cut-off) for 12 h at 4°C with three buffer changes [27]. Antibody concentration was determined spectrophotometrically at 280 nm using an extinction coefficient of 1.36 for 1 mg/mL immunoglobulin G (IgG). Final yields were expressed in mg/mL.

### Evaluation of polyclonal antisera

#### Indirect ELISA

Antibody titers were assessed using the IDEXX BVDV Total Antibody Kit (IDEXX, USA). Sera were tested at dilutions of 1:1, 1:25, 1:50, and 1:100, with appropriate controls. Optical density (OD) was read at 450 nm, and results were expressed as sample-to-positive (S/P) ratios. Cutoff criteria were ≤0.20 = negative, ≥0.30 = positive, and 0.21–0.29 = doubtful. Doubtful results were retested.

#### SDS-PAGE

Purity of antisera was analyzed by SDS-PAGE under reducing and non-reducing conditions. Samples were mixed with a loading buffer (2% SDS), boiled (10 min), and 20 μL was loaded onto a 10% polyacrylamide gel (Shandong Welldone Environmental New Materials Co., Ltd. Qingdao, Shandong, China). A pre-stained molecular weight marker (10–180 kDa) was included. Electrophoresis was run in Tris-glycine (Sigma Aldrich, catalog number T4904, Singapore) buffer at 120 V. Gels were stained with Coomassie Brilliant Blue G-250 (Thermo Fisher Scientific Inc. Waltham, MA 02451, United States) and destained in 10% methanol/10% acetic acid [28].

#### IPMA

MDBK cells were seeded into 96-well plates (1 × 10^5^ cells/mL, 50 μL/well) in EMEM with 5% FBS, fungizone (1 μg/mL), and gentamycin (20 μg/mL). After overnight incubation at 37°C with 5% CO_2_, wells were inoculated with 25 μL BVDV; controls received distilled water. After 48 h, monolayers were washed with PBS–0.05% Tween-20, fixed with 35% acetone in PBS containing 0.02% bovine serum albumin (BSA; South Pacific Sera, Timaru, New Zealand), and dried at 37°C.

Cells were incubated sequentially with rabbit anti-BVDV-1a polyclonal antibody (1:10 dilution, 60 min), biotin-labeled secondary antibody (10 min), and peroxidase-conjugated streptavidin (15 min), with PBS-Tween washes between steps. Antigen–antibody complexes were visualized with H_2_O_2_/3,3′-diaminobenzidine for 1 h, followed by hematoxylin counterstaining (3 min) [29, 30].

## RESULTS

### Purification of polyclonal antisera

The purification process yielded polyclonal antisera with a total protein concentration of 40.33 mg/mL, as determined by UV spectrophotometry at 280 nm.

### Antibody titer determination

Antibody titers were assessed using indirect ELISA according to the manufacturer’s guidelines, in which samples with an S/P ratio ≥0.3 were considered positive. In this assay, the negative control OD was 0.24, and the positive control OD was 1.58, giving a denominator of 1.34 for the S/P calculation. The detailed ELISA results are summarized in Table 1.

**Table 1:** ELISA antibody titer determination of the tested serum based on OD values and S/P ratios across serial dilutions.

Dilution	OD (sample)	S/P ratio	Interpretation
1:1	2.72	1.85	Strong positive
1:25	2.25	1.50	Strong positive
1:50	2.07	1.37	Strong positive
1:100	1.63	1.04	Strong positive

ELISA = Enzyme-linked immunosorbent assay, OD = Optical density, S/P = Sample-to-positive.

### SDS-PAGE analysis

**Figure 1:**
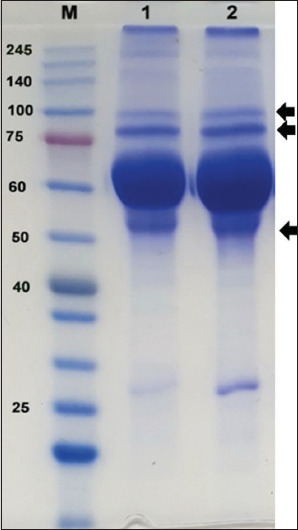
SDS-PAGE analysis of purified rabbit polyclonal anti-bovine viral diarrhea virus-1a antibody. Lane M: molecular weight marker. Bands were observed at approximately 53, 75, and 100 kDa under reduced and non-reduced conditions, respectively. The arrow indicates antibody-related protein bands. SDS-PAGE = Sodium dodecyl sulfate–polyacrylamide gel electrophoresis.

The purity of the rabbit polyclonal anti-BVDV-1a antibody was analyzed by SDS-PAGE. Distinct protein bands were observed at approximately 53, 75, and 100 kDa under both reducing and non-reducing conditions (Figure 1). These bands correspond to antibody-related proteins, with possible additional protein components present in the sample matrix.

### IPMA

**Figure 2:**
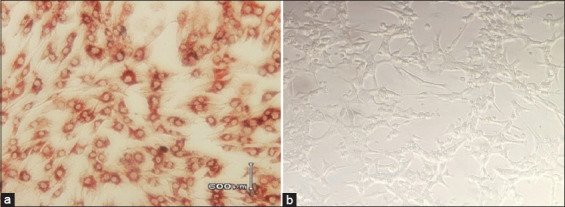
(a and b) Immunoperoxidase monolayer assay staining results. The brownish cytoplasmic coloration observed in Madin–Darby bovine kidney cells indicates viable BVDV actively replicating within the cells (a). The lack of observable cytopathic effects suggests that the BVDV strain used has a non-cytopathic biotype (b). BVDV = Bovine viral diarrhea virus.

The specificity of the polyclonal antisera was further confirmed using IPMA. MDBK cells infected with BVDV demonstrated clear brown cytoplasmic staining, indicative of a positive antigen–antibody reaction (Figure 2a). In contrast, uninfected MDBK cells showed no staining (Figure 2b), confirming the specificity of the antisera. Quantitative assessment using ImageJ software (developed by Wayne Rasband at the National Institutes of Health) measured the cytoplasmic staining intensity, further validating the reactivity and specificity of the produced polyclonal antibodies against BVDV.

## DISCUSSION

### Antibody concentration and immunization effectiveness

The antibody concentrations obtained in this study were measured to assess the effectiveness of the immunization strategy. The average antibody concentration was 40.33 mg/mL, indicating a strong immune response to the antigen. Antibodies produced by the immune system in response to an immunogen are crucial reagents extensively used in various applications, techniques, and instruments. Successful antibody production depends on the immunogenicity of the antigen and its ability to elicit a robust immune response. According to Zhang *et al*. [31], immunogenicity is influenced not only by the antigen properties but also by the immunization protocol and the selection of the host animal, which plays a critical role in determining the efficiency and yield of antibody production. These results demonstrate that the immunization protocol employed in this study effectively induced a measurable and substantial antibody response, confirming the suitability of the antigen and host system.

### Comparison with previous studies

Comparable studies yield in the range of single digit to low tens of milligrams: for instance, Nasiri *et al*. [24] recovered ~12 mg of purified rabbit IgG, Thomas *et al*. [32] obtained ~8 mg/mL of rabbit serum under optimized conditions, and Majidi *et al*. [33] achieved ~8 mg in 1 mL buffer with ~98% purity. These benchmarks indicate that our yield is within or even beyond typical ranges, supporting the effectiveness of our purification approach.

### Antibody titer determination

The persistence of S/P ratios well above the positivity threshold, even at a 1:100 dilution, indicates a high antibody titer in the tested serum. Such strong reactivity across serial dilutions reflects a robust immune response to the target antigen, confirming that the antibodies are present at sufficient levels for reliable detection. Similar studies by Majidi *et al*. [33] and Ridpath *et al*. [34] have reported BVDV antibody titers remaining positive up to dilutions of 1:64–1:128 in naturally infected cattle, whereas experimentally immunized rabbits often show detectable reactivity only up to 1:50 [35]. In comparison, the strong positivity observed up to 1:100 dilution suggests that the immune response generated was not only strong but also within the upper range of reported titers. This finding supports the suitability of the produced antibodies for diagnostic use and assay development.

### Importance of host animal selection

The choice of host animal is a critical factor in polyclonal antibody production, as it influences the total antibody yield, ease of sample collection, and antigen immunological compatibility. In this study, 3-month-old New Zealand white rabbits were selected as the experimental model due to their manageable body size, ease of handling, ability to produce substantial amounts of IgG, and resilience to multiple blood withdrawals without adverse effects [36]. Rabbits can yield approximately 250 mg of polyclonal antibody from 50 mL of blood, making them a suitable choice for this study [37]. Furthermore, IgG production is reported to significantly increase at 3 months of age, highlighting this period as the optimal time for immunization [36, 38].

### Application of ELISA in diagnostics

ELISA is a well-established, antibody-based method widely employed for field applications, particularly for determining disease prevalence in unvaccinated herds. A positive ELISA result indicates the presence of infection within a herd, making it a crucial tool in disease surveillance and control programs. Given the high antibody titers observed in this study, the developed polyclonal antibodies hold promise for further applications in diagnostic assays and potential therapeutic interventions. Future studies could focus on refining immunization protocols, optimizing antigen formulations, and evaluating the longevity and specificity of the generated antibodies to enhance their utility in veterinary and medical research.

### SDS-PAGE characterization of antibodies

To further assess the purity of the rabbit polyclonal anti-BVDV-1a antibody, SDS-PAGE analysis was performed. The results (Figure 1) revealed distinct bands corresponding to Ig components. A major band was observed at approximately 53 kDa, which is consistent with the IgG heavy chain, while additional bands at ~75 kDa and ~100 kDa may represent intact IgG molecules or antibody dimers, respectively. These findings confirm the successful production of polyclonal antibodies against BVDV-1a in rabbits.

Previous studies have reported similar electrophoretic patterns. For instance, rabbit IgG typically yields bands around 50–55 kDa (heavy chain) and ~25 kDa (light chain) under reducing conditions, whereas intact IgG or dimeric forms may appear between 70 and 100 kDa in non-reducing gels [32, 33]. Therefore, the presence of these characteristic bands in this study supports the successful purification of functional IgG.

The presence of additional faint bands suggests the presence of non-IgG proteins, which are common in crude polyclonal antibody preparations. However, the strong ELISA reactivity observed in this study demonstrates that the functional IgG fraction was present in sufficient purity and concentration to ensure effective antigen binding. Further purification strategies, such as affinity chromatography, could be applied in future studies to increase antibody homogeneity and reduce background reactivity.

Overall, the SDS-PAGE analysis validates that the immunization and purification protocols employed in this study were effective for generating functional rabbit polyclonal anti-BVDV-1a antibodies suitable for diagnostic assay development.

### Validation by IPMA

In this study, we complemented ELISA and SDS-PAGE characterization of the produced polyclonal antibody with an IPMA to validate its diagnostic potential for BVDV. IPMA is an accessible immunocytochemical method that is often preferred in virology laboratories for detecting viral antigens in infected cell monolayers due to its operational simplicity, minimal reliance on expensive instrumentation, and relatively high sensitivity [39].

Our findings showed that BVDV-infected MDBK cells developed clear brown cytoplasmic staining, whereas uninfected cells remained unstained. This differential signal directly confirms that the polyclonal antibody binds specifically to BVDV antigens *in situ*, in their intracellular compartments, without appreciable non-specific binding. The absence of visible CPEs in the stained cultures supports the conclusion that the viral isolate is of the non-cytopathic biotype, which replicates without overt cell destruction (consistent with the literature on BVDV) [35]. Thus, our IPMA result not only validates antibody specificity but also provides biologically relevant insights into the viral phenotype.

To make the assay more objective and quantitative, we employed ImageJ to measure staining intensity within defined cytoplasmic regions. The statistically significant elevation of mean staining intensity (infected vs. control, p < 0.01) underscores the robust and reproducible binding of the antibody. The use of image analysis enhances the rigor of the assay by reducing the subjectivity inherent in visual scoring and improving reproducibility across operators and experiments.

### Comparative context of IPMA

The comparative literature shows that IPMA has broad application in virus diagnostics. For example, Direksin *et al*. [39] used IPMA for detecting antiswine influenza antibodies; Afayoa *et al*. [40] compared IPMA with PCR and hemagglutination for African swine fever detection (finding IPMA to be a viable complementary method); Liang *et al*. [41] developed an IPMA for detecting swine hepatitis E virus based on open reading frame 3-expressing cell lines; and Haegeman *et al*. [42] applied IPMA for lumpy skin disease virus serodiagnosis. IPMA has served as a middle ground between high-throughput ELISA and more complex molecular assays in many of these settings.

Previous studies by Haegeman *et al*. [42] and Tao *et al*. [43], which compared polyclonal and monoclonal antibodies in IPMA, have found comparable sensitivity and specificity under various conditions. For instance, Deregt and Prins [44] developed an IPMA using monoclonal antibody pools to detect BVDV types 1 and 2, demonstrating that a well-selected monoclonal IPMA can sometimes outperform polyclonal IPMA in reading ease and sensitivity, though retesting often equalized performance (with the polyclonal version reaching similar sensitivity). This suggests that with optimization (antibody concentration, incubation times, blocking, and substrate development), the polyclonal antibody in our work could be brought to parity with monoclonal-based systems.

One of the key advantages of polyclonal antibodies is their ability to recognize multiple epitopes on the viral antigen. This broad epitope coverage can confer resilience to antigenic variation or minor conformational changes in viral proteins that might otherwise reduce the binding of more narrowly focused monoclonal antibodies. In the context of BVDV, where strain diversity and antigenic drift are recognized challenges to diagnostics and vaccination [45], a robust polyclonal reagent may be particularly valuable. Moreover, in field or clinical samples surveillance, where viral loads and epitope exposure may differ from controlled culture conditions, the polyclonal antibody’s flexibility may improve diagnostic yield.

### Limitations of the study

Although this preliminary study successfully demonstrated antibody production and characterization, several constraints should be considered when interpreting the results:

1. Small sample size and statistical power: The study used a limited number of experimental animals, which constrains statistical power and generalizability. Variability may lead to over- or underestimation of antibody concentrations or titers with few biological replicates.
2. Pooling of antibody samples: Antibodies were pooled across animals to mask individual variations in immune responses. This pooling prevents the assessment of how each rabbit’s immune system responded and may obscure weaker or stronger responders.
3. Lack of statistical analysis of individual variability: Due to pooling and the small sample size (n), a detailed statistical comparison between individual animals (e.g., variance, confidence intervals) was not possible. Thus, we cannot comment on the consistency of the immune response across different subjects.
4. Potential bias in antibody characterization: Average (pooled) values may make the antibody appear more effective than individual responses. Moreover, some typical background or non-specific protein contaminants in polyclonal preparations could affect purity and binding specificity, which pooling might further obscure.
5. Reproducibility challenges: Variability in biological response due to genetic differences, handling, or immunization schedule may influence the results of this study. Without individual data and with pooled antibody, replicability (especially in different laboratories or with different animals) may be reduced.

These limitations should be taken into account when interpreting the study’s results. Future studies with larger sample sizes, individual animal analyses, and standardized antibody preparations are recommended to validate and extend these findings.

### Novelty and contribution

However, despite the limitations inherent in this preliminary study, the successful development of high-titer polyclonal antisera against an Indonesian isolate of BVDV-1a, characterized through ELISA and SDS-PAGE, and validated by IPMA, represents a significant advancement in BVDV research and diagnostics in Indonesia, where such resources have been previously unavailable.

## CONCLUSION

This preliminary study successfully produced high-titer polyclonal antisera against a local Indonesian BVDV-1a isolate and demonstrated their functional characterization. The purified antisera exhibited an average antibody concentration of 40.33 mg/mL, as determined by SDS-PAGE, which revealed distinct immunoglobulin bands at ~53, 75, and 100 kDa. ELISA confirmed strong antibody titers, maintaining positivity up to 1:100, whereas IPMA validated the antisera’s specificity by distinct cytoplasmic staining in BVDV-infected MDBK cells. Collectively, these findings confirm that the immunization protocol and purification strategy employed were effective in generating functional polyclonal antibodies with robust reactivity and specificity.

The development of locally tailored antisera addresses a critical gap in BVDV diagnostics in Indonesia, where reliance on imported ELISA kits designed from non-local strains may compromise detection accuracy. These antisera provide an essential biological resource for improving diagnostic assays, supporting surveillance programs, and guiding disease control strategies. In a broader context, they contribute to livestock health management, food security, and economic resilience in cattle production systems.

A major strength of this study lies in the combination of immunological (ELISA), biochemical (SDS-PAGE), and cellular (IPMA) approaches, providing a multidimensional validation of antibody functionality. The high antibody yield achieved here exceeds typical values reported in similar experiments, further demonstrating the efficiency of the chosen antigen preparation and immunization protocol.

While the findings are promising, further work is needed to expand the sample size, assess antibody performance across individual animals, and test diagnostic accuracy using diverse field samples, such as serum, milk, and tissue biopsies. Comparative studies with monoclonal antibodies and molecular methods (e.g., RT-PCR) will be necessary to establish sensitivity and specificity benchmarks. Future refinement of purification methods, such as affinity chromatography, could improve antibody homogeneity. Ultimately, these antisera may be incorporated into region-specific ELISA kits or adapted for point-of-care diagnostic tools in the field.

In conclusion, this study provides the first report of high-titer polyclonal antisera against a local Indonesian BVDV-1a strain. Despite limitations such as a small sample size and pooled sera, the results demonstrate the feasibility of generating region-specific immunological reagents to strengthen diagnostic capacity. With further validation and development, these antisera hold significant potential for advancing BVDV surveillance, supporting national control programs, and contributing to the broader One Health framework by safeguarding animal health, agricultural productivity, and food security.

## AUTHORS’ CONTRIBUTIONS

SUK: Rabbits’ management before and during the study. HW: Antigen preparation, polyclonal antibody purification, and enzyme-linked immunosorbent assay. RW, SS, and DA: Rabbit immunization and blood sampling. MHW: Conducted SDS-PAGE. SUK, HW, RW, SS, DA, and MHW: Drafted and revised the manuscript. All authors have read and approved the final version of the manuscript.
